# Work Outcomes after Intensity-Modulated Proton Therapy (IMPT) versus Intensity-Modulated Photon Therapy (IMRT) for Oropharyngeal Cancer

**DOI:** 10.14338/IJPT-20-00067.1

**Published:** 2021-06-25

**Authors:** Grace L. Smith, Shuangshuang Fu, Matthew S. Ning, Diem-Khanh Nguyen, Paul M. Busse, Robert L. Foote, Adam S. Garden, Gary B. Gunn, Clifton D. Fuller, William H. Morrison, Gregory M. Chronowski, Shalin J. Shah, Lauren L. Mayo, Jack Phan, Jay P. Reddy, James W. Snider, Samir H. Patel, Sanford R. Katz, Alexander Lin, Nasiruddin Mohammed, Roi Dagan, Nancy Y. Lee, David I. Rosenthal, Steven J. Frank

**Affiliations:** 1 Department of Radiation Oncology, University of Texas MD Anderson Cancer Center, Houston, TX, USA; 2 Department of Health Services Research, University of Texas MD Anderson Cancer Center, Houston, TX, USA; 3 University of California Riverside School of Medicine, Riverside, CA, USA; 4 Department of Radiation Oncology, Massachusetts General Hospital, Harvard Medical School, Boston, MA, USA; 5 Department of Radiation Oncology, Mayo Clinic and Mayo Clinic School of Medicine and Science, Rochester, MN, USA; 6 Department of Radiation Oncology, University of Maryland School of Medicine, Baltimore, MD, USA; 7 Department of Radiation Oncology, Mayo Clinic and Mayo Clinic School of Medicine and Science, Phoenix, AZ, USA; 8 Willis-Knighton Proton Therapy Center, Shreveport, LA, USA; 9 Department of Radiation Oncology, University of Pennsylvania, Philadelphia, PA, USA; 10 Northwestern Medicine Chicago Proton Center, Warrenville, IL, USA; 11 Department of Radiation Oncology, University of Florida College of Medicine, Gainesville, FL, USA; 12 Department of Radiation Oncology, Memorial Sloan Kettering Cancer Center, New York, NY, USA

**Keywords:** oropharyngeal cancer, proton therapy, work, productivity, patient-reported outcomes

## Abstract

**Purpose:**

We compared work outcomes in patients with oropharyngeal cancer (OPC), randomized to intensity-modulated proton (IMPT) versus intensity-modulated photon therapy (IMRT) for chemoradiation therapy (CRT).

**Patients and Methods:**

In 147 patients with stage II-IVB squamous cell OPC participating in patient-reported outcomes assessments, a prespecified secondary aim of a randomized phase II/III trial of IMPT (n = 69) versus IMRT (n = 78), we compared absenteeism, presenteeism (i.e., the extent to which an employee is not fully functional at work), and work productivity losses. We used the work productivity and activity impairment questionnaire at baseline (pre-CRT), at the end of CRT, and at 6 months, 1 year, and 2 years. A one-sided Cochran-Armitage test was used to analyze within-arm temporal trends, and a χ^2^ test was used to compare between-arm differences. Among working patients, at each follow-up point, a 1-sided Wilcoxon rank-sum test was used to compare work-productivity scores.

**Results:**

Patient characteristics in IMPT versus IMRT arms were similar. In the IMPT arm, within-arm analysis demonstrated that an increasing proportion of patients resumed working after IMPT, from 60% (40 of 67) pre-CRT and 71% (30 of 42) at 1 year to 78% (18 of 23) at 2 years (*P* = 0.025). In the IMRT arm, the proportion remained stable, with 57% (43 of 76) pre-CRT, 54% (21 of 39) at 1 year, and 52% (13 of 25) working at 2 years (*P* = 0.47). By 2 years after CRT, the between-arm difference between patients who had IMPT and those who had IMRT trended toward significance (*P* = 0.06). Regardless of treatment arm, among working patients, the most severe work impairments occurred from treatment initiation to the end of CRT, with significant recovery from absenteeism, presenteeism, and productivity impairments by the 2-year follow-up (*P* < 0.001 for all). Higher magnitudes of recovery from absenteeism (at 1 year, *P* = 0.05; and at 2 years, *P* = 0.04) and composite work impairment scores (at 1 year, *P* = 0.04; and at 2 years, *P* = 0.04) were seen in patients treated with IMPT versus those treated with IMRT.

**Conclusion:**

In patients with OPC receiving curative CRT, patients randomized to IMPT demonstrated increasing work and productivity recovery trends. Studies are needed to identify mechanisms underlying head and neck CRT treatment causing work disability and impairment.

## Introduction

Rising costs of cancer care have prompted a pressing need for “value” in cancer treatment decisions [1–4]. The challenge to achieve “high-value” radiation oncology care is particularly relevant in oropharyngeal cancer (OPC), a head and neck cancer that uses radiation treatment as the mainstay for cure [5]. The current standard for curative therapy in OPC requires 6 to 7 weeks of radiation directly targeting the soft tissues and lymph nodes of the head and neck, concurrent with chemotherapy. Because of this intensive, multimodality treatment approach for head and neck cancer, conventional treatment is associated with toxicities that can be symptomatically severe and impair a patient's daily function: acutely, mucositis, thick secretions, dehydration, poor nutrition, and requirement of narcotic pain medications, and, in the long term, xerostomia, dysphagia, dysgeusia, lymphedema, trismus, esophageal stricture, and dependency on a feeding tube [6–10]. It is not surprising that the health care costs of patients with head and neck cancer in survivorship are among the costliest in the United States [11].

Evidence suggests that patients with OPC and other head and neck cancers are also personally vulnerable to economic burdens related to the illness and disability after their intensive therapy course and subsequent toxicities [12–15]. Patients with head and neck cancer frequently miss or lose work during radiation treatment, with prior studies reporting between 40% and 60% of patients still failing to return to work in long-term survivorship [12, 13]. Relatedly, patients with head and neck cancer report personal financial distress or “financial toxicity” [16]. A recent single-center study [17] found that about one third of patients with head and neck cancer had moderate to catastrophic financial toxicity with radiation treatment.

Therefore, treatment strategies that address clinical toxicities in patients with OPC may also lead to benefits in functional recovery, ability to return to work, and downstream financial toxicity. To address the severe clinical toxicities associated with current OPC treatment, one strategy in head and neck radiation is intensity-modulated proton therapy (IMPT) because IMPT seeks to improve clinical outcomes through reducing radiation-associated toxicity when compared with conventional intensity-modulated photon therapy (IMRT). Moreover, IMPT takes advantage of the unique physical properties of protons (Bragg peak), allowing minimal radiation exit doses and sparing surrounding healthy tissues from resultant toxicity [18, 19]. Prior observational studies of patients with OPC have demonstrated that IMPT was associated with lower risks of dry mouth, swallowing difficulties, damaged taste, and feeding-tube dependence, with equivalent cancer survival rates when compared with conventional IMRT [20–22].

Therefore, with differences in radiation toxicity and long-term symptom profiles, treatment with IMPT versus IMRT could potentially demonstrate an effect on downstream work and financial toxicity outcomes [14]. Despite a growing appreciation for the need to compare the effect of different radiation treatment strategies on those outcomes, it is unknown whether IMPT versus IMRT may lead to different work outcomes in patients with OPC. Accordingly, we sought to compare work and productivity outcomes, prospectively and longitudinally measured by the validated work productivity and activity impairment (WPAI) instrument in patients with OPC who received either IMPT or IMRT in a randomized trial setting.

## Materials and Methods

### Data Source and Study Sample

This study reports results from a prespecified secondary outcome analysis of a randomized phase II/III clinical trial (NCT01893307) [23], which was approved by the University of Texas MD Anderson Cancer Center Institutional Review Board. Between 2014 and 2018, 192 patients provided consent and were enrolled in our phase II trial of IMPT versus IMRT for definitive chemoradiation (CRT) for stage II–IVB [24] squamous cell carcinoma of the oropharynx. Eligibility criteria included patients who were older than 18 years with histologically documented cancer and an Eastern Cooperative Oncology Group performance status of 0 to 2 and, for women, who had a negative pregnancy test. Randomization to IMPT versus IMRT treatment arms was stratified by receipt of induction chemotherapy, human papillomavirus (HPV)/p16 status, and smoking status (≤ 5 pack years versus > 5 pack years). The primary outcome of the phase II trial was treatment toxicity, and the primary phase III randomized trial is ongoing. Of enrolled patients, we excluded patients who withdrew before randomization (N = 2; 1%) and who withdrew after randomization (N = 3; 2%). This analysis includes 147 patients (79%) who participated in longitudinal survey assessment of patient-reported outcomes; 40 patients elected not to participate in the survey assessments (**Supplemental Figure S1**).

### Treatment

In the randomized trial, all patients received concurrent chemotherapy (eg, cisplatin 40 mg/m^2^ weekly), prescribed at the discretion of the medical oncologist. The total prescribed radiation dose to gross disease was 70 Gy radiobiologic equivalent (GyRBE) at 2.12 GyRBE/fraction and, for low-risk microscopic disease, was 57 GyRBE at 1.72 GyRBE/fraction, for a total of 33 fractions. Target volume definitions were in accordance with the International Commission on Radiation Units and Measurements report 50 [25] and 62 [26]. Prescribed doses and margins to target volumes were consistent between proton and photon plans, which encompassed ≥ 95% of the planning target volume and could be normalized to the 93% to 98% prescription isodose line. In IMPT, intensities of all pencil beams (“beamlets”) of multiple beams were simultaneously optimized to achieve the desired balance between healthy tissue and target coverage. Pencil beams of individual beams were not required to reach the distal edge of the target volume. Low-dose regions and dose heterogeneity in the target per beam were compensated for with pencil beams from other directions. Robust optimization addressed range-sensitive IMPT plans to ensure IMPT plan validity.

### Work and Productivity Outcome Measures and Covariates

Patient-reported work outcomes were prospectively measured with the validated WPAI questionnaire [27] as a prespecified trial aim. The WPAI questionnaire was completed by patients at baseline (pre-CRT), at the end of CRT, and at 6 months, 1 year, and 2 years after therapy. The WPAI questionnaire assessed various dimensions of work productivity and activity impairment. Working status was evaluated with a yes/no question that asked patients to report whether they were working for pay in the prior week (within the prior 7 days). Among those who were working, the following dimensions of work productivity were additionally assessed in the questionnaire: (1) absenteeism, which quantified a patient's reported hours of missed work relative to his or her total typical work hours; (2) presenteeism, which quantified a patient's perceived change in work quality compared with his or her usual work quality; and (3) productivity impairment, which compositely quantified absenteeism and presenteeism [28]. Absenteeism, presenteeism, and productivity impairment were scored on a scale of 0 to 100, based on the scoring calculation approach of Reilly et al [28], with higher numbers (closer to 100) representing recovery from work impairment and lower numbers (closer to 0) representing worsening work impairment levels, for ease of interpretation. For analysis at each time point, each patient's change (recovery) in their score was calculated as: Δ = *Score at follow-up time point* − *Score pre-CRT*, so that Δ > 0 represented recovery of work impairment over time, whereas Δ < 0 represented a persistent worsening of absenteeism, presenteeism, or work productivity as measured by a composite work impairment score over time. Patients' baseline sociodemographic characteristics and disease and tumor covariates were obtained from medical records and baseline trial data abstractions.

### Statistical Analysis

Baseline characteristics were compared between patients who were randomized to IMPT and to IMRT treatment arms. Continuous variables were compared by *t* test, and categorical variables were compared by the χ^2^ test. For the outcome of employment (patients working and returning to work), we used the Cochran-Armitage test for within-arm temporal trends for a change in the proportion of patient employment during follow-up in each of the groups of patients treated with IMPT and with IMRT. For between-arm comparisons of the outcome of employment, we tested univariate difference in proportions of patient employment at each time point by χ^2^ tests. We tested a generalized linear mixed model with binomial distribution to identify whether there were significant longitudinal changes in the proportion of patients working.

For outcomes of work impairment, we used a one-sided Wilcoxon rank-sum test with nonnormal distribution to compare Δ scores for absenteeism, presenteeism, and work productivity, as measured by a composite work impairment score, assessing the change in score at each follow-up point (6 months, 1 year, and 2 years) from the pre-CRT score. Additionally, we tested unadjusted and adjusted generalized linear mixed models for those scores to identify significant longitudinal changes in absenteeism, presenteeism, and productivity scores over time, as well as to identify predictors of longitudinal changes. In multivariate analysis, covariates with associations of *P* < 0.25 in univariate analysis and clinical significance were used to identify parsimonious models. Regarding missingness, the general linear mixed models provided unbiased estimates of effects. We alternatively tested a last-observation carry-forward approach for sensitivity analysis. All analyses were conducted with SAS Enterprise Guide software (version 7.11; SAS Institute, Cary, North Carolina), and *P* values < 0.05 for 1- and 2-tailed tests were considered statistically significant.

## Results

### Patient Characteristics

In 147 patients, mean (SD) age was 59 (9.1) years, and 88% (n = 129) were men, 83% (n = 122) were stage IVA to IVB, and 96% (n = 141) had HPV-positive results. At baseline, 73% (n = 107) of patients were employed (not retired) before cancer diagnosis. In patients randomized to receive IMPT versus IMRT, baseline sociodemographic and clinical characteristics were well balanced, without significant differences (Table 1). During the follow-up period, the response rate (the number of patients with a scoreable questionnaire, divided by the number participating in the patient-reported survey outcomes assessments) was 56% (82 of 147) at 6 months, 55% (81 of 147) at 1 year, and 33% (48 of 147) at 2 years.

**Table 1.** Patient characteristics by treatment arm.

**Characteristic**	**IMPT, No. (%), N = 69**	**IMRT, No. (%), N = 78**	***P*** **value**
Age, mean (SD), y	59.4 (9.1)	58.7 (9.1)	0.67
Male	64 (92.8)	65 (83.3)	0.08
White race	63 (91.3)	71 (91.0)	0.95
Cancer stage per AJCC [24]			0.58
II/III	12 (17.4)	11 (14.1)	
IVA/IVB	57 (82.6)	67 (85.9)	
Induction chemotherapy	8 (11.6)	12 (15.4)	0.50
HPV^+^	66 (95.7)	75 (96.2)	0.88
Baseline employment			
Retired	17 (24.6)	22 (28.2)	0.92
Primary insurance			1.00
Employer-based	35 (50.7)	40 (51.3)	
Medicare	19 (27.5)	22 (28.2)	
Other	11 (15.9)	12 (15.4)	

Abbreviations: IMPT, intensity-modulated proton therapy; IMRT, intensity-modulated photon therapy; AJCC, *AJCC Cancer Staging Manual*; HPV, human papillomavirus**.**

### Employment and Return-to-Work Outcomes

At pre-CRT baseline, before beginning active treatment, only 58% (83 of 143) of patients overall were actively working, with relatively little change at 65% (31 of 48) by the 2-year follow-up. This overall stable longitudinal pattern for the entire cohort was confirmed in the mixed model (*P* = 0.32 for time covariate). However, in the within-arm analysis, patients treated with IMPT, a significantly increased proportion resumed working over time, from 60% (40 of 67) pre-CRT and 71% (25 of 35) at 1 year to 78% (18 of 23) at 2 years (1-sided Cochran-Armitage test for a trend of *P* = 0.025). In the within-arm analysis of the IMRT arm, the proportions remained stable, with 57% (43 of 76) at pre-CRT, 54% (21 of 39) at 1 year, and 52% (13 of 25) at 2 years (one-sided Cochran-Armitage test for trend *P* = 0.47). At 2 years after CRT, the between-arm comparison between patients in IMPT and IMRT treatment arms approached significance (χ^2^ test, *P* = 0.06; Figure, Table 2).

**Figure.**
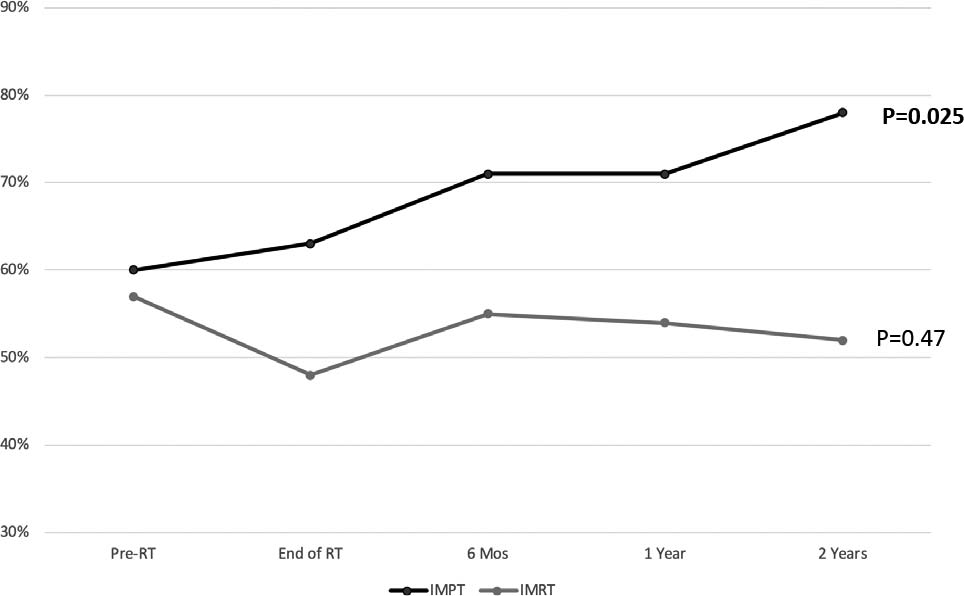
Temporal trends in the proportion of patients working during radiation treatment and survivorship, tested within the group of patients treated with intensity-modulated proton therapy (IMPT) and those treated with intensity-modulated photon therapy (IMRT). The within-group trend was tested with a 1-sided Cochran-Armitage test for trend.

**Table 2.** Between-arm comparison of the proportion of patients working during radiation treatment and survivorship.

**Randomization arm**	**Pre-CRT**	**End of CRT**	**6 mo**	**1 y**	**2 y**
IMPT, n = 69					
Response rate, No. (%)	67 (97)	43 (62)	35 (51)	42 (61)	23 (33)
Working per response, No. (%)	40 (60)	27 (63)	25 (71)	30 (71)	18 (78)
IMRT, n = 78					
Response rate, No. (%)	76 (97)	40 (51)	47 (60)	39 (50)	25 (32)
Working per response, No (%)	43 (57)	19 (48)	26 (55)	21 (54)	13 (52)
Between-arm comparison *P* value^a^	0.71	0.16	0.14	0.10	0.06

Abbreviations: CRT, chemoradiation therapy; IMPT, intensity-modulated proton therapy; IMRT, intensity-modulated photon therapy.

a χ^2^ test.

### Work and Productivity Impairment

In the entire sample of patients, there was a distinct pattern of work impairment and recovery. During cancer treatment, patients experienced their nadir of absenteeism, presenteeism, and productivity scores (worst median score for each of those measures). Median scores for each of those measures also significantly and steadily improved (recovered) during the follow-up period from 6 months to 2 years after CRT (all *P* < 0.001 for time covariates in each mixed-effects growth model for outcomes, with the parameter estimates indicating improvement over time for absenteeism, presenteeism, and productivity impairment scores. **Supplemental Tables S1-S3**).

Higher magnitudes of recovery, or improvement, in absenteeism scores were seen for patients randomized to IMPT versus IMRT. Complete recovery would be indicated by Δ = + 100, and no change would be Δ = 0. In patients treated with IMPT, the median improvement in absenteeism scores at 1 year was Δ = + 40 (interquartile range [IQR] + 71 and 0) versus Δ = + 8 (IQR + 35 and 0) in patients treated with IMRT (1-sided Wilcoxon rank-sum, *P* = 0.054). At 2 years, median improvement in absenteeism scores was Δ = + 40 (IQR + 75 and 0) in patients treated with IMPT versus Δ = + 0 (IQR + 38 and 0) in patients treated with IMRT (1-sided Wilcoxon rank-sum, *P* = 0.04. **Table 3**).

**Table 3.** Mean and median changes in work and productivity impairment (WPAI) scores during survivorship among working patients with oropharyngeal cancer (OPC) by treatment arm. Positive change (Δ) in absenteeism and composite work impairment scores represent an improvement from before chemoradiation therapy (pre-CRT) baseline score; negative change in scores represents a worsening of impairment from pre-CRT treatment baseline score.

**Randomization arm**	**Absenteeism**					**Composite work impairment**			
	**Pre-CRT**	**End of CRT**	**6 mo**	**1 y**	**2 y**	**End of CRT**	**6 mo**	**1 y**	**2 y**
IMPT arm									
Mean (SD), Δ	−	−19 (33)	+11 (49)	+38 (39)	+41 (37)	−22 (31)	+9 (50)	+38 (37)	+40 (38)
Median (IQR), Δ	−	0 (0, −43)	0 (+24, −45)	+40 (+71, 0)	+40 (+75, 0)	−5 (+39, −47)	0 (+46, −20)	+38 (+70, 0)	+46 (+70, 0)
IMRT arm									
Mean (SD), Δ	−	−19 (28)	+19 (42)	+21 (37)	+21 (27)	−23 (32)	+16 (41)	+23 (38)	+20 (29)
Median (IQR), Δ	−	0 (0, −33)	+8 (+45, 0)	+8 (+35, 0)	0 (+38, 0)	0 (0, −47)	+9 (+52, 0)	+23 (+48, 0)	+9 (+35, 0)

Abbreviations: IQR, interquartile range; IMPT, intensity-modulated proton therapy; IMRT, intensity-modulated photon therapy.

Similarly, higher magnitudes of recovery in productivity measured by composite work impairment scores were seen for patients randomized to IMPT versus IMRT. At 1 year, the improvement in composite work impairment scores was Δ = + 38 (IQR + 70 and 0) in patients treated with IMPT versus Δ = + 23 (IQR + 48 and 0) in patients treated with IMRT (1-sided Wilcoxon rank-sum, *P* = 0.05). At 2 years, the improvement was Δ = + 46 (IQR + 70 and 0) in patients treated with IMPT versus Δ = + 9 (IQR + 35 and 0) in patients treated with IMRT (1-sided Wilcoxon rank-sum *P* = 0.04. **Table 3**).

There were no differences in presenteeism recovery between treatment arms. In generalized linear mixed models, other predictors of significantly better work impairment recovery over time were more-favorable baseline scores (eg, starting with less absenteeism, presenteeism, and work impairment levels before cancer treatment), more time after treatment, and younger age (**Supplemental Tables S1–S3**).

## Discussion

Patients with OPC who undergo definitive therapy with CRT are at high risk for financial toxicity and missed or lost work associated with the intensive multimodality therapy [15]. Moreover, with the epidemiology of OPC incidence and prevalence shifting toward an increasing representation by younger, working-aged patients [29], these disease trends underscore the population effect of missed or lost work after treatment. There is a need to identify optimal CRT strategies that help to preserve work and productivity, as well as to minimize the economic burden of disease and treatment for patients and society.

Results from this analysis help to address that knowledge gap. Our analysis provided a concerning new insight into the overall longitudinal pattern of the frequency of return to work—with little improvement in the overall frequency of return to work in patients with OPC—only about 60% (n = 88) of patients, even at 2 years of follow-up. Furthermore, among those who did maintain or return to work, many reported substantial work impairments (absenteeism, presenteeism, and impaired productivity). The most marked severity of those work impairments occurred during active CRT through 6 months after CRT, although with significant recoveries in scores for work impairment between 1 and 2 years after treatment.

The randomized design of this study with work and productivity as prespecified endpoints additionally allowed for a novel direct comparison of patients randomized to IMPT versus IMRT treatment arms. In patients randomized to the IMPT arm, results showed a trend toward an increased proportion of recovery and return to work to near pre-CRT levels of employment. At 1 year, 71% (n = 49) of patients randomized to IMPT were working, whereas, at the same follow-up time point, 54% (n = 42) who were randomized to IMRT were working, and that proportion remained unchanged among IMRT patients over time. Results further demonstrated a trend toward IMPT patients experiencing greater recovery from absenteeism and greater work productivity during long-term follow-up. At 1-year and 2-year times, the difference in absenteeism and composite work impairment scores for IMPT versus IMRT patients approached or achieved a “minimally important difference” in scores, calculated as 0.5 ×SD or 19 points. Although the underlying causal mechanisms for these differences in productivity by treatment arm are yet unknown, it is possible that differences in toxicities and symptom burdens seen on prior observational studies of patients with OPC who receive proton treatment could contribute to differences in recovery of function, recovery that may be effective enough to influence long-term disability. Recent observational studies of oropharyngeal or nasopharyngeal cancer, which included long-term survivors, identified the significance of fatigue, neurobehavioral dysfunction, and disability as contributing to work loss in these populations [30, 31]. Therefore, the potential acute, subacute, and long-term toxicity profile benefits of IMPT could have a role in mitigating work outcomes. Ultimately, these findings generate an additional provocative hypothesis that, in patients with OPC, a strategy that uses IMPT could have the potential to increase recovery of illness-related costs and decrease patient economic burdens compared with IMRT, “value-based” oncology outcomes that merit additional investigation [1–4, 32–34].

The main limitation of this study is that detailed data on the underlying reason(s) why participants did or did not return to work were not available. In addition to the potential contribution of toxicity as an underlying barrier to return to work, alternatively, prior studies have identified that, after cancer treatment, the significance of a diagnosis of cancer may have an important psychologic effect, from which patients reframe their life circumstances and consider alternative life plans, which may trigger intentional change (discontinuation) of employment [35]. In contrast, the decision to return to work may also, in fact, be motivated by other financial concerns patients face, such as needing to pay direct medical-care costs. Finally, not all patients who were randomized to treatment participated in the survey, and any biases in response related to unblinded treatment could not be controlled for, although there were not significant differences between known characteristics of respondents treated with IMPT or IMRT. Additional study is also needed to define and quantify the subset of patients who discontinue employment who are still seeking a job (true unemployment). Most patients in this trial were enrolled from a single center, almost all were HPV^+^; therefore, external validation of these results is warranted. If validated, findings may help affect health policy, payer policy, and employer policy levels to support and expand use of proton therapy for OPC.

## Conclusions

In patients with OPC who receive curative CRT, a substantial proportion fail to return to work after treatment, and among those who do work, there are sustained impairments demonstrated by absenteeism and lower productivity, even during survivorship. Our analysis demonstrated a trend toward patients randomized to IMPT more frequently remaining as workers throughout treatment and during long-term follow-up. Moreover, among those who were able to remain working, patients randomized to IMPT also more often showed sustained recovery of productivity. Future studies are needed to identify the mechanisms underlying treatment-related factors contributing to work disability and impairment after curative head and neck CRT.
