# Supplementary material for: Work Outcomes after Intensity-Modulated Proton Therapy (IMPT) versus Intensity-Modulated Photon Therapy (IMRT) for Oropharyngeal Cancer
Source: Int J Part Ther. 2021 Jun 25;8(1):319–27. doi: 10.14338/IJPT-20-00067.1 (PMC8270077; doi:10.14338/IJPT-20-00067.1)

eFigure 1. Patient enrollment in the parent randomized clinical trial and number of voluntary participants in the patient reported work outcomes survey, by treatment group

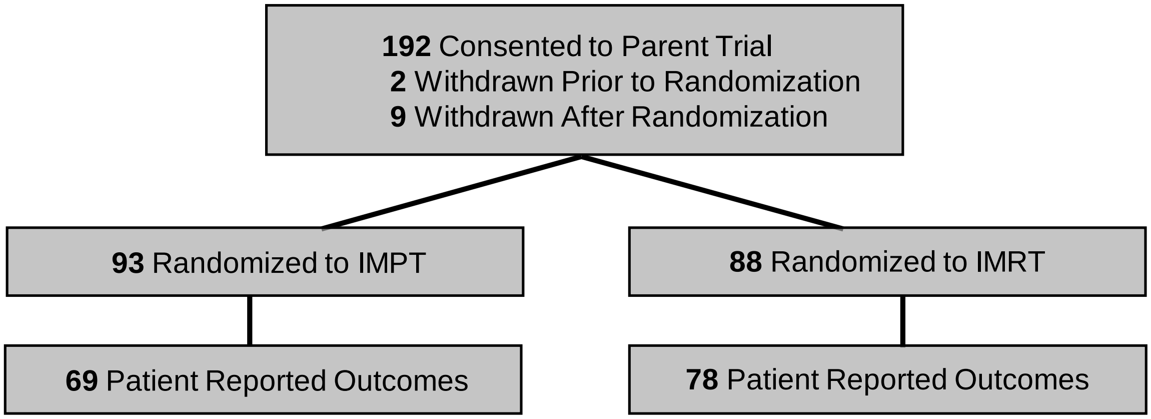

Supplement: Supplementary file 1 [file ijpt-08-01-21_s01.pdf]
