# Supplementary material for: Work Outcomes after Intensity-Modulated Proton Therapy (IMPT) versus Intensity-Modulated Photon Therapy (IMRT) for Oropharyngeal Cancer
Source: Int J Part Ther. 2021 Jun 25;8(1):319–27. doi: 10.14338/IJPT-20-00067.1 (PMC8270077; doi:10.14338/IJPT-20-00067.1)
Supplement: Supplementary file 2 [file ijpt-08-01-21_s02.doc]

**eTable 1. Multivariate generalized linear mixed model: Predictors of longitudinal change in absenteeism score**

|  | **Estimate** | **Standard Error** | **DF** | **p-value** |
| --- | --- | --- | --- | --- |
| **Intercept** | 0.09 | 0.15 | 68 | 0.56 |
| **IMPT vs. IMRT** | 0.02 | 0.03 | 582 | 0.61 |
| **Baseline Score** | 0.25 | 0.05 | 582 | **<0.0001** |
| **Time in Follow-up** | -0.02 | 0.002 | 74 | **<0.0001** |
| **Age at Diagnosis** | 0.004 | 0.002 | 582 | 0.07 |
| **Male Gender** | -0.003 | 0.07 | 582 | 0.97 |
| **Non-White Race** | 0.10 | 0.07 | 582 | 0.13 |
| **Stage (IVA/IVB)** | -0.03 | 0.04 | 582 | 0.51 |
| **Induction Chemo** | -0.08 | 0.06 | 582 | 0.16 |
| **HPV Positive** | 0.07 | 0.09 | 582 | 0.46 |

*Abbreviations: IMPT intensity modulated proton therapy; IMRT intensity modulated photon therapy; DF degrees of freedom; HPV human papilloma virus*

**eTable 2. Multivariate generalized linear mixed model: Predictors of longitudinal change in presenteeism score**

|  | **Estimate** | **Standard Error** | **DF** | **p-value** |
| --- | --- | --- | --- | --- |
| **Intercept** | -0.20 | 0.13 | 66 | 0.12 |
| **IMPT vs. IMRT** | 0.001 | 0.03 | 579 | 0.96 |
| **Baseline Score** | 0.30 | 0.07 | 579 | **<0.0001** |
| **Time in Follow-up** | -0.01 | 0.001 | 72 | **<0.0001** |
| **Age at Diagnosis** | 0.01 | 0.002 | 579 | **0.002** |
| **Male Gender** | 0.03 | 0.06 | 579 | 0.57 |
| **Non-White Race** | 0.07 | 0.05 | 579 | 0.19 |
| **Stage (IVA/IVB)** | 0.002 | 0.04 | 579 | 0.96 |
| **Induction Chemo** | 0.06 | 0.05 | 579 | 0.24 |
| **HPV Positive** | 0.06 | 0.08 | 579 | 0.46 |

*Abbreviations: IMPT intensity modulated proton therapy; IMRT intensity modulated photon therapy; DF degrees of freedom; HPV human papilloma virus*

**eTable 3. Multivariate generalized linear mixed model: Predictors of longitudinal change in work productivity impairment score**

|  | **Estimate** | **Standard Error** | **DF** | **p-value** |
| --- | --- | --- | --- | --- |
| **Intercept** | -0.05 | 0.17 | 68 | 0.77 |
| **IMPT vs. IMRT** | 0.007 | 0.04 | 582 | 0.87 |
| **Baseline Score** | 0.30 | 0.05 | 582 | **<0.0001** |
| **Time in Follow-up** | -0.02 | 0.002 | 74 | **<0.0001** |
| **Age at Diagnosis** | 0.007 | 0.003 | 582 | **0.005** |
| **Male Gender** | -0.0004 | 0.08 | 582 | 1.00 |
| **Non-White Race** | 0.11 | 0.07 | 582 | 0.11 |
| **Stage (IVA/IVB)** | -0.03 | 0.05 | 582 | 0.59 |
| **Induction Chemo** | -0.01 | 0.06 | 582 | 0.83 |
| **HPV Positive** | 0.11 | 0.10 | 582 | 0.30 |

*Abbreviations: IMPT intensity modulated proton therapy; IMRT intensity modulated photon therapy; DF degrees of freedom; HPV human papilloma virus*
